# The Faces of Generative AI: Predictors of FACES

**DOI:** 10.1007/s12663-025-02547-8

**Published:** 2025-05-07

**Authors:** Christopher R. Wolfe, Mackenzie M. Blazek, Paige A. Renschler, Lauren M. Lucina, Deepak G. Krishnan

**Affiliations:** 1 Department of Psychology, Miami University, Oxford, OH USA; 2 Division of Oral & Maxillofacial Surgery, Department of Surgery, University of Cincinnati, Cincinnati, OH USA

**Keywords:** DALL·E, Generative artificial intelligence, Faces, Visual stimuli, Maxillofacial anomalies

## Abstract

**Background:**

Facial Appearance as Core Expression Scales (FACES) was designed to assess maxillofacial surgery patients’ perceptions of their faces. We used FACES to study ratings of non-patient participant’s own faces, and faces produced by the generative AI application DALL·E.

**Materials and Methods:**

DALL·E was used to generate 16 photo-realistic faces of males and females aged 20, 30, 40, and 50 of differing apparent ethnicities. Four stem descriptions were used for four sets of four images, for example “The face of a 40-year-old male (or female) of mixed South American and African ancestry wearing something dark in the style of a professional photo portrait.” This was followed by descriptions taken from all seven FACES items, such as “the face is (not) like I want others to see me.” Image generation instructions differed only in “not” being used for half of the images.

**Results:**

Participants (n = 333) rated images using FACES to test the hypothesis that FACES can distinguish between faces generated by positively and negatively worded instructions. That hypothesis was confirmed for all 8 image pairs.

**Conclusion:**

We also found that Rosenberg Self-Esteem and State Self Esteem Scale scores predicted FACES ratings of participants’ own faces. However, DALL·E was unable to depict realistic maxillofacial anomalies.

## Introduction

Perhaps there is nothing more closely tied to one’s identity than their own face. Much of the human experience surrounding faces relates to how faces are recognized or processed. Facial recognition has been found to be processed as a whole, rather than by parts [1]. Additionally, facial recognition is sensitive to the context in which it is being processed [1]. However, beauty itself is found to be relatively resistant to external factors and context [1]. It is found, though, that judgments of morality based on faces is influenced by external factors, which is one reason why facial recognition can have large contextual influences [2]. Facial attractiveness can be measured and tracked in many ways; one approach is eye tracking. It has been found that greater eye movement is correlated with viewing a more attractive face [3]. Developmentally, eye tracking is found to increase throughout infancy and directly correlate with increases in facial recognition and bias [4]. Thus, psychological research on faces is important, particularly in an era when realistic faces are being produced by generative Artificial Intelligence (AI).

Recently, AI image generation by text-to-image models, has impressed the public with its sophistication and realism. Whereas traditional AI algorithms have relied on structured data for model building and information processing, machine learning techniques have fundamentally altered AI research in recent years [5]. Machine learning is crucial as it allows systems to acquire new information and expand their knowledge, judgments, and conclusions through experience and data without being explicitly programmed to do so beforehand [6]. More advanced generative AI algorithms have evolved to process data in natural language, enabling the generation of images from text. These algorithms have evolved to where convoluted neural networks and recurrent neural networks have gained the ability to analyze and produce images, audio, and video [5].

Natural Language Processing (NLP) has been important in the development of chatbots as the use of NLP techniques allows these programs to understand and interpret human language input [6]. OpenAI ^©^ is an artificial intelligence company that has released several generative AI applications including DALL·E and ChatGPT. DALL·E, along with many other chatbots, uses NLP input to create imagery in multiple styles, including photorealistic imagery, paintings, and emoji [6]. Any user can input text, such as a descriptive caption, into DALL·E, prompting it to generate images based on the input received. Given the power of these tools, research on the ability of generative AI, particularly DALL·E, to process text descriptions of faces has theoretical and practical implications.

Facial Appearance as Core Expression Scale (FACES) was developed to assess how individuals perceive themselves and how well it represents their ideal self. This instrument can help to identify and reduce disparities in expectations of facial surgical patients who may not have the same perceptions as their surgeons. This instrument has many uses including the ability to quantify facial satisfaction the patient experiences, ability to compare surgical techniques, and the ability to identify patients who may be unlikely to perceive benefits from surgery.

Patients answer seven questions about how much they agree or disagree with how much those statements apply to their face today by moving a slider from 0 to 100. These seven statements include: my face is pretty or handsome, my face is like I want it to be, my face is like I want others to see me, my face is appealing, my face is attractive, my face is likable, my face is like my ideal self. This FACES score is an average of these seven responses, with higher numbers corresponding to a more positive view of one’s own face. This set of seven items was highly reliable in previous research with Cronbach’s α = 0.94 [7]. Research by Wolfe et al. indicates that in a non-surgical population most individuals rated themselves between 40 and 80, and less than 3% would rate themselves lower than 20 [7]. In another study, participants rated their own face as well as several other facial images using the FACES instrument. Eight of the pictures shown were of 4 patients who had undergone maxillofacial surgery with before and after surgery pictures. The results from this study suggested that FACES items are sensitive to the results of surgical interventions and validated this instrument in detecting predicted differences with high sensitivity of about 0.77 standard deviations [7]. A third study investigated individual differences with participants rating their own faces and completing the Body Image Avoidance Questionnaire, Rosenberg Self-Esteem, and State Self Esteem Scale, and each predicted FACES outcomes with R^2^ = 0.43 [8]. This indicates a strong relationship between self-esteem and people’s perceptions of their own faces as measured with the FACES instrument.

The purpose of this study was to investigate the potential for DALL·E 2 to create useful stimuli for medical and psychological research on faces using FACES. If the text interface of DALL·E is sensitive to the characteristics of human faces captured with the FACES instrument, then textual descriptions of faces based on those characteristics should produce visual stimuli (pictures of faces) that participants rate differently depending on whether the generated faces were generated with positively-worded instructions (e.g. “the face is like I want others to see me”) on negatively-worded instructions (e.g. “the face is **not** like I want others to see me”). The hypothesis was that faces generated by DALL·E using positively worded instructions will score significantly higher on FACES than those that are worded negatively by the addition of “not” before the same positively worded statements. This hypothesis was tested across a range of instructions to DALL·E for apparent age, ethnicity, and gender.

## Methods

*Study design/sample*: In order to answer the research question, a prospective cohort study was designed. The Internal Review Board (IRB) approved the protocol in accordance with 45 CFR 46.10 on 11/06/2022 letter number 02121r. The study population was composed of a sample of Miami University student volunteers enrolled in Psychology 112. Students participating in the research received course credit for taking part in the research. The inclusion criterion was being at least 18 years of age, exclusion criterion was being younger than 18 years of age. Participants were recruited through the SONA online subject recruitment site and provided informed consent after both oral and written descriptions of the study with text approved by the IRB to fulfil a requirement in a psychology course.

**Fig. 1:**
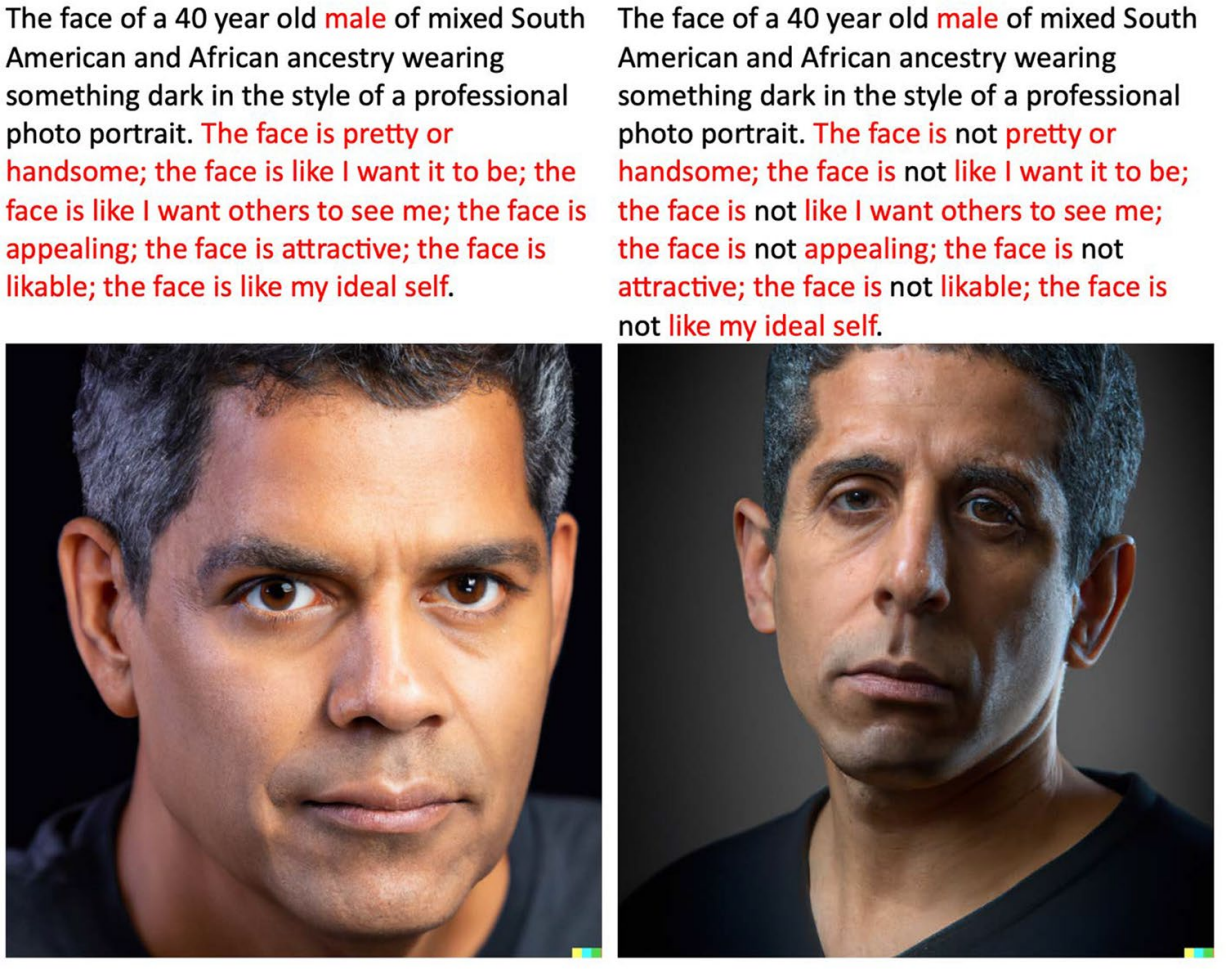
Examples of DALL·E 2 Generated Faces

Faces were created by giving written descriptions to DALL·E 2 at OpenAI (https://labs.openai.com/). The approach was to start with basic stem descriptions that were common to four images and covered ages of 20, 30, 40, and 50 years old, with half describing males and half females. The descriptions were designed to cover a range of ethnicities. In each case the images were described as photo-realistic in the style of a portrait, with dark colored clothes to maintain the focus on the faces. Added to these stems, half of the descriptions were the seven items from the FACES instrument and the other half were negatively worded by adding the word “not” to each of the seven faces items [7]. For example, the two faces in Fig. 1 were created with the description, starting on the left, “The face of a 40 year old male of mixed South American and African ancestry wearing something dark in the style of a professional photo portrait. The face is pretty or handsome; the face is like I want it to be; the face is like I want others to see me; the face is appealing; the face is attractive; the face is likable; the face is like my ideal self.” The image on the right side of Fig. 1 was created using the same description except that the word “not” was added to each FACES item. Participants then rated each face using the FACES instrument [7]. Appendix A presents all 16 of the images created for Study 1 along with the text prompts used to create them, and the results of participant ratings. Finally, participants were asked demographic questions.

**Fig. 2:**
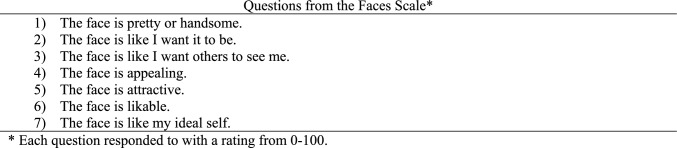
Faces scale questions for each AI generated face

*Data Collection Methods*: Participants were seated in in the first author’s laboratory in front of a desktop computer and run individually or in sets of two to four students seated at separate tables facing away from one another. Study participants responded using a slider set at the middle of a scale from 0 = Strongly Disagree to 100 = Strongly Agree for each of the seven FACES questions (see Fig. 2). Participants had to move the slider to proceed but could return it to the midpoint if they choose. For the DALL·E faces task, the order of the 16 items were presented in a unique, random order differing for each participant. Participants were then thanked and debriefed using language approved by the IRB.

*Data Analyses*: Data were analyzed using the SAS package JMP 15.2.1 with paired t-tests being used to compare the mean composite FACES ratings of positively and negatively rated computer-generated faces. Cohen’s *d* was used as a measure of effect size, and Cronbach’s α was used to assess the reliability of the 7 FACES items in contributing to the composite FACES score. The α level was set at *p* ≤ 0.05.

## Results

The final study sample was composed of 333 Miami University undergraduates. There were 258 who self-described as female, 72 male, and 3 self-described as other. The mean age was 18.7 years (SD ± 1.0). Considering race and ethnicity, 17 (5.2%) identified as Hispanic, and 280 (84.6%) said they were White, 15 (4.5%) Black/African American, 15 (4.5%) Mixed Ethnicity, 16 (4.8%) Asian, 3 preferred to self-describe, and 2 were Native American.

Positive faces had a mean FACES score of 51.0 (SD ± 11.3) which was significantly higher than negative faces with a mean of 30.8 (SD ± 12.0), t(332) = 37.39, *p* < 0.0001, Cohen’s d = 1.7. This effect held for male faces, with positive male faces having an average FACES score of 47.0 (SD ± 12.2) and negative faces having a mean of 30.8 (SD ± 12.0), t(332) = 29.37, *p* < 0.0001 as well as female faces, with positive male faces having a mean FACES rating of 55.0 (SD ± 13.4) and negative faces having a mean of 30.8 (SD ± 12.0), t(332) = 34.40. As seen in Appendix A, the faces generated through positive wording scored significantly higher than those created with negative wording for all 8 pairs of images.

To assess covariance among FACES items while rating faces different than participants own faces, we applied Cronbach’s α to FACES items for 50-year-old male faces created using positive and negative wording. These faces were chosen as being the most different from most participants, who were 77% female with a mean of 18.7 years old. The positive 50-year-old DALL·E faces had a mean of 39.5 (SD ± 15.8) with Cronbach’s α = 0.882 with all FACES items and lower with any items removed. The negative 50-year-old DALL·E faces had a mean of 34.9 (SD ± 14.7) with Cronbach’s α = 0.868 with all FACES items and lower with any items removed. These results suggest that all seven FACES items contribute to overall ratings of DALL·E faces.

Turning to FACES ratings of participants own faces, participants gave their own faces a mean rating of 62.0 (SD = 17.9) with Cronbach’s alpha = 0.95 and lower with any items removed. This replicates the high reliability of FACES and suggest that participants were generally positive about their own faces. The second hypothesis was tested by correlations between FACES ratings of participant’s own faces, and measures of self-esteem. The Pearson correlation between FACES and SSES was 0.66, *p* < 0.01, and − 0.61 for Rosenberg Self Esteem, *p* < 0.01. The hypothesis that self-esteem is a predictor of FACES ratings was supported.

## Discussion

Written instructions to DALL·E to create faces were highly sensitive to items in the FACES instrument, resulting in large and robust differences in faces created using positive and negative wording (Cohen’s d = 1.7). This suggests that DALL·E is a useful tool for creating faces as stimuli in research related to issues such as attractiveness and representation of faces. It also provides further evidence of the robustness of FACES, extending beyond ratings of people’s own faces and neutral faces to faces differing sharply in apparent age, sex, and ethnicity. FACES may be used to address research questions about other people’s faces as well as our own.

We had sought to use DALL·E to create faces with various maxillofacial anomalies. However, these efforts were unsuccessful. We used a number of different stems and descriptions of maxillofacial anomalies, and none produced images of faces accurately depicting those anomalies. For example, Appendix B shows the results of the description “The face of a 30 year of a female with unilateral hyperplasia of the mandible of mixed European and indeterminant ethnicity wearing something dark in the style of a professional photo portrait.” Yet the image does not depict a person with hyperplasia of the mandible. This limitation appears to be not entirely accidental. We asked ChatGPT 3.5 (also created by OpenAI), “Why can’t I use DALL·E to create faces with maxillofacial anomalies?” As shown in Appendix C, the answer includes limited training data, abstract interpretation, complexity of medical conditions, and ethical considerations. Thus, in its current iteration DALL·E is unsuitable for creating stimuli in psychological studies of reactions to maxillofacial anomalies.

These data suggest that importance of maxillofacial surgery (the initial impetus for the creation of FACES) is far from “skin deep” as it has direct effects on fundamental aspects of well-being. Moreover, when surgeons and patients have very different perceptions of the success of surgery, these findings suggest that deeper issues of body image and self-esteem may be in play. It appears that exploring these issues may be valuable in cases of deep dissatisfaction on the part of patients. Additional research is needed to explicate the relationship between self-esteem and our perceptions of our own faces. One important limitation of the current work is that we recruited undergraduate students in the U.S. who were not patients to serve as participants. FACES should be tested with clinical patients, particularly before and after maxillofacial surgery recruited in several countries. The results of the current work suggest that research of this kind is warranted and overdue.

## Appendix A

Descriptions used to create pictures of faces with DALL·E and the resulting images for males and females ages 20, 30, 40, and 50 years old. Mean FACES ratings, standard deviations, and the results of t-tests are reported for each pair.

## Appendix B

Limitations of DALL·E: unsuccessful at creating faces with maxillofacial anomalies.

## Appendix C

November 11, 2023 dialogue with ChatGPT 3.5 on creating faces with maxillofacial anomalies.

Researcher: Why can’t I use DALL·E to create faces with maxillofacial anomalies?

ChatGPT: I apologize for any confusion in my previous responses. DALL·E, as a generative model, has the capability to generate images based on textual descriptions, and it can indeed create a variety of images, including faces with certain features or anomalies. However, there are several reasons why using DALL·E for specific medical or anatomical conditions, such as maxillofacial anomalies, might be challenging:

1. *Limited training data*: DALL·E is trained on a diverse dataset, but the training data might not comprehensively cover every possible medical condition or anatomical anomaly. Therefore, the model might not accurately capture the nuances of specific maxillofacial anomalies.
2. *Abstract interpretations*: DALL·E is known for its creative and abstract interpretations of textual prompts. When generating images based on text, the model may prioritize artistic or imaginative aspects rather than strict anatomical accuracy.
3. *Complexity of medical conditions*: Maxillofacial anomalies can be highly complex and diverse. Representing them accurately requires a deep understanding of medical imaging and anatomy, which may go beyond the scope of DALL·E’s training data.
4. *Ethical considerations*: Generating images of individuals with specific medical conditions raises ethical considerations. Using AI in a medical context requires careful consideration of privacy, consent, and the potential impact on individuals with the conditions in question.

While DALL·E can be a creative tool for generating diverse visual content, it may not be the most suitable choice for creating highly accurate and realistic representations of medical conditions. For precise medical imaging or anatomical illustrations, it’s recommended to rely on specialized tools, medical imaging databases, or collaborate with healthcare professionals who can provide accurate information and expertise.
